# How Gas Cylinders Are Made

**Published:** 1892-04

**Authors:** 


ARTICLE V.

### HOW GAS CYLINDERS ARE MADE.

The supply of compressed gas in metal cylinders has now assumed the proportions of an important industry, more especially since it was found possible, by the Brin process, to obtain oxygen direct from the atmosphere.

The industry is not exactly a new one, for carbon dioxide and nitrous oxide (the latter for the use of dentists) have been supplied in a compressed state for many years. Now, with the creation of the modern amateur photographer, who can make lantern slides, and the more general adoption of the optical lantern for the purposes of demonstration and amusement, there has arisen a demand for the limelight such as was never experienced before, and as the limelight is dependent upon the two gases, hydrogen and oxygen, for its support, these gases are now supplied in large quantities commercially. At first the gas cylinders were made of wrought iron; they were cumbrous and heavy, and the pressure of the inclosed gas was so low that a receptacle to hold only ten feet was a most unwieldy concern. But times have changed, and a cylinder of about the same size, that half the weight, is now made to hold four times the quantity of gas at the enormous initial pressure of 1,800 pounds on every square inch. This means the pressure which an ordinary locomotive has to withstand multiplied by twelve. The change is due to improved methods of manufacture and to the employment of mild steel of special quality in lieu of the wrought iron previously employed. The cylinders are now made without joint or seam, and the process of manufacture is most interesting. A short time ago we had an opportunity of watching the various necessary operations involved in making these cylinders at the Birmingham works of Messrs. Taunton, Delamard & Co., by whose courtesy we were enabled to make notes of the progress.

Beginning with the raw material we were shown a disk of metal measuring thirty inches in diameter and three-quarters of an inch in thickness. From such a "blank" a cylinder destined to hold 100 feet of compressed gas can be constructed, and the first operation is to heat the "blank" in a furnace, and afterwards to stamp it into cup-like form. To all intents and purposes this represents the end of a finished cylinder, but it is far too bulky to form the end of one of the size indicated; indeed, it in reality contains enough metal to make the entire vessel. By a series of operations it is now heated and drawn out longer and longer, while its thickness diminishes and its diameter grows less. These operations are carried out by means of a number of hydraulic rams, which regularly decrease in size. The plunger may be compared to a finger and the cylinder to a glove, while the well may represent a hole into which both are thrust in order to reduce the thickness of the glove. With huge tongs the cylinder, fresh from the furnace, is placed in position, but just before the plunger presses into the red hot cup, one of the workmen empties into the latter a litter water, so as to partially cool the bottom and prevents its being thrust out by the powerful plunger. Oil is also used plentifully, so that as the plunger works slowly down the red hot mass, it is surrounded by smoky flames. It presently forces the cylinder into the well, and when the end of the stroke is reached, a stop piece is inserted through an opening in the upper part of the well, so as to arrest the edge of the cylinder while the reverse action of drawing out the plunger is proceeded with. Directly the finger is drawn out of the glove—in other words, immediately the plunger is raised out of the cylinder—the latter drops down below with a heavy thud, still in a red hot condition.

This operation of hot drawing is repeated again and again in rams of diminishing size until the cylinder assumes the diameter and length required. This hot drawing leaves the surface of the metal marked with longitudinal lines, not unlike the glacier scratches on a rock, albeit they are straighter and more regular. But the next operation not only obliterates these markings, and gives the metal a smooth surface like that of polished silver, but it also confers upon the material a homogeneity which it did not before possess, and without which it would never bear the pressure which it is destined to withstand when finished. This operation consists in a final application of the hydraulic ram while the metal remains perfectly cold, instead of red hot, as in the previous cases.

As the result of these various hydraulic operations, we have a perfectly formed cylinder closed at one end, and we now follow it into another department of the works, when its open end is once more brought in a furnace to a red heat. The object of this is to make the metal soft, while the shoulder and neck of the vessel are formed. To accomplish this, the heated open end of the cylinder is laid horizontally upon a kind of semicircular cradle, and is held there by tongs handled by two men. Another workman places over the open end a die, and while the cylinder is slowly turning around in its cradle, two sledge hammers are brought down with frequent blows upon the die, closing in the end of the cylinder, but leaving a central hole. Further operations reduce the opening still more until it is closed together, and a projection is formed. This projection is now bored through, and the cylinder is ready for testing.

The cylinder is submitted to a water test; the liquid being forced in until the gauge shows a pressure of two tons to the square inch. Cylinders have been known to give away under this ordeal, but without any dangerous consequences. The metal simply rips up, making a report at the moment of fracture as loud as a gun. The wonderful strength of the metal employed may be gauged by the circumstance that the walls of the cylinder designed to hold 100 feet of gas are only five-sixteenths of an inch in thickness.

During the manufacture of the cylinder, as we have already indicated, much oil is used, and so far as steel can be saturated with that fluid—in the popular sense—the metal is in that state. It is essential that this oil should be completely got rid of, and this is carefully done before the cylinder is charged with gas. Previous to such charging, the vessel has to be fitted with its valve. Of these valves there are three kinds; known respectively as the

Brin, the Birmingham, and the Manchester. Each has its admirers, but we cannot here discuss their individual merit.

The charging of these cylinders is brought about by a powerful pump having three cylinders so arranged that the compressed contents of the first cylinder are still further compressed in the second, and still more highly in the third. The filling of a 100 ft. cylinder occupies about half an hour.—*Photographic News*.

